# Cerium Dioxide–Dextran Nanocomposites in the Development of a Medical Product for Wound Healing: Physical, Chemical and Biomedical Characteristics

**DOI:** 10.3390/molecules29122853

**Published:** 2024-06-15

**Authors:** Ekaterina V. Silina, Natalia E. Manturova, Olga S. Ivanova, Alexander E. Baranchikov, Elena B. Artyushkova, Olga A. Medvedeva, Alexey A. Kryukov, Svetlana A. Dodonova, Mikhail P. Gladchenko, Ekaterina S. Vorsina, Maria P. Kruglova, Oleg V. Kalyuzhin, Yulia G. Suzdaltseva, Victor A. Stupin

**Affiliations:** 1 I.M. Sechenov First Moscow State Medical University (Sechenov University), Moscow 119991, Russia; marykruglova@live.ru (M.P.K.); kalyuzhin@list.ru (O.V.K.); 2 Pirogov Russian National Research Medical University, Moscow 117997, Russia; manturovanatali@yandex.ru (N.E.M.); stvictor@bk.ru (V.A.S.); 3 Frumkin Institute of Physical Chemistry and Electrochemistry, Russian Academy of Science, Moscow 119071, Russia; runetta05@mail.ru; 4 Kurnakov Institute of General and Inorganic Chemistry, Russian Academy of Sciences, Moscow 119991, Russia; a.baranchikov@yandex.ru; 5 Kursk State Medical University, Karl Marx Str., 3, Kursk 305041, Russia; eartyushkova@mail.ru (E.B.A.); medvedevaoa@kursksmu.net (O.A.M.); krukovaa@kursksmu.net (A.A.K.); dodonovasa@kursksmu.net (S.A.D.); mgladchenko@yandex.ru (M.P.G.); vorsinaes@kursksmu.net (E.S.V.); 6 Vavilov Institute of General Genetics, Russian Academy of Sciences, Gubkin Str., 3, Moscow 119333, Russia; yu_suzdaltseva@mail.ru

**Keywords:** wound healing, nanocomposite, nanoparticles, nanodrug, dextran, nanocerium, cerium dioxide, polysaccharide, polysaccharide–metal complexes, fibroblasts, gas chromatography, regeneration, antimicrobial activity

## Abstract

Purpose of the study: the creation of a dextran coating on cerium oxide crystals using different ratios of cerium and dextran to synthesize nanocomposites, and the selection of the best nanocomposite to develop a nanodrug that accelerates quality wound healing with a new type of antimicrobial effect. Materials and methods: Nanocomposites were synthesized using cerium nitrate and dextran polysaccharide (6000 Da) at four different initial ratios of Ce(NO_3_)_3_x6H_2_O to dextran (by weight)—1:0.5 (Ce0.5D); 1:1 (Ce1D); 1:2 (Ce2D); and 1:3 (Ce3D). A series of physicochemical experiments were performed to characterize the created nanocomposites: UV-spectroscopy; X-ray phase analysis; transmission electron microscopy; dynamic light scattering and IR-spectroscopy. The biomedical effects of nanocomposites were studied on human fibroblast cell culture with an evaluation of their effect on the metabolic and proliferative activity of cells using an MTT test and direct cell counting. Antimicrobial activity was studied by mass spectrometry using gas chromatography–mass spectrometry against *E. coli* after 24 h and 48 h of co-incubation. Results: According to the physicochemical studies, nanocrystals less than 5 nm in size with diffraction peaks characteristic of cerium dioxide were identified in all synthesized nanocomposites. With increasing polysaccharide concentration, the particle size of cerium dioxide decreased, and the smallest nanoparticles (<2 nm) were in Ce2D and Ce3D composites. The results of cell experiments showed a high level of safety of dextran nanoceria, while the absence of cytotoxicity (100% cell survival rate) was established for Ce2D and C3D sols. At a nanoceria concentration of 10^−2^ M, the proliferative activity of fibroblasts was statistically significantly enhanced only when co-cultured with Ce2D, but decreased with Ce3D. The metabolic activity of fibroblasts after 72 h of co-cultivation with nano composites increased with increasing dextran concentration, and the highest level was registered in Ce3D; from the dextran group, differences were registered in Ce2D and Ce3D sols. As a result of the microbiological study, the best antimicrobial activity (bacteriostatic effect) was found for Ce0.5D and Ce2D, which significantly inhibited the multiplication of *E. coli* after 24 h by an average of 22–27%, and after 48 h, all nanocomposites suppressed the multiplication of *E. coli* by 58–77%, which was the most pronounced for Ce0.5D, Ce1D, and Ce2D. Conclusions: The necessary physical characteristics of nanoceria–dextran nanocomposites that provide the best wound healing biological effects were determined. Ce2D at a concentration of 10^−3^ M, which stimulates cell proliferation and metabolism up to 2.5 times and allows a reduction in the rate of microorganism multiplication by three to four times, was selected for subsequent nanodrug creation.

## 1. Introduction

Public health issues are among the most important issues in the modern world. Among the many issues requiring medical care, trauma, including wounds, ranks first in terms of frequency and severity [1,2,3]. Ubiquitous health advertising and sports, especially extreme sports, have played a negative role, providing an increase in the number of somatic and skin wounds in the most physically active part of the human population, those aged 14–35 years old [4]. A special place is occupied by gunshot wounds, the number of which continues to increase proportionally both in civil society and in zones of armed military conflicts [5,6,7]. The important differences of gunshot wounds are the combination of the lesion with extensive skin lesions and the obligatory contamination of the wounds with defeaters, a wide range of aerobic and anaerobic microorganisms. The situation is greatly aggravated by the decreasing antimicrobial activity of antimicrobial drugs and increasing antibiotic resistance and has become one of the major problems in modern medicine and pharmacology. Rapid mutations of microorganisms, outpacing the speed of development of new antimicrobial drugs, slowly but surely ensure the transition of this problem from the category of issues requiring resolution to a state close to disaster. The “epidemic of antibiotic-resistant microorganisms” that has spread everywhere, especially in intensive care units, where the fight against bacterial complications is one of the top priorities, cannot be considered otherwise [8,9,10,11,12,13,14].

Regeneration in skin injuries is the second most important but the most frequently addressed problem. Acute skin damage in everyday life is a routine situation, but chronic ulcers in patients with diabetes mellitus, tissue arterial ischemia, varicose veins, and postthrombotic syndrome are a heavy burden on family and state budgets [15,16,17,18]. Great hopes were pinned on mesenchymal stem cells. The biological theories that were being put forward were impressive due to their coherence and completeness [19,20,21,22]. The results obtained in laboratories and vivariums gave hope of a breakthrough in healthcare and successful promotion of these technologies in clinical practice. Unfortunately, the breakthrough did not happen, and the scientific medical world began to be conquered by a new idea—the idea of using nanomaterials that exhibit unexpectedly strong biological effects that could be used to treat some somatic diseases and skin lesions [23,24].

Interest in nanoparticles in medicine, which first emerged in the middle of the last century, grew rapidly as the results of experiments with metal nanoparticles, especially those with variable valence, became available. The confirmed mechanisms of drug delivery directly to tumor cells using nanomaterials, which reduced the toxic load and improved the treatment outcome of cancer patients, further ensured the interest of the medical community in nanotechnology [25,26,27,28,29,30,31].

One promising metal oxide is cerium dioxide. In the last five years, according to PubMed data from 2019 to 2024, there were 7066 papers published on nanoceria, almost double the number of publications in the previous 5 years. Most of the studies investigating the biological effects of nanoceria have shown its redox activity as well as regenerative and even antimicrobial and pro-regenerative effects [32,33,34,35,36,37,38,39]. There are many problems in the synthesis of new groups of nanodrugs that have not been encountered before. For example, the biological activity of nanoceria depends not only on the chemical purity of the product. The shape of the nanoceria crystal and its properties vary depending on the method of its synthesis, changes in the pH of the medium, etc. [34,39,40,41,42]. Similarly, the same dependencies occur with different nanoparticle coatings [41,42,43]. Coatings of cerium nanoparticles with various substances, most often of plant origin, are a necessity because they prevent the aggregation of nanocrystals, which leads to a significant decrease in the total area of crystals, which comes into direct contact with the environment. This aggregation leads to a decrease in or disappearance of biological effects, which are illustrated by many researchers when using properly prepared nanoceria sols [44,45,46,47]. Natural polysaccharides (agar-agar, alginate, pullunan, chitosan, fucoidan, dextran, and pectin) are often used as coatings for metal nanoparticles [44,48,49]. Focusing on the world literature, dextran, which has long been used in medicine for several indications, has performed well in many biological studies with nanoparticles [50,51,52,53,54,55]. It is known that dextran polysaccharide is a branched glucose polymer with an average chain mass ranging from 3 to 20000 kDa, which is synthesized by acetic acid bacteria from sucrose. In our studies, we also chose dextran as more accessible and showing high tropism to cerium dioxide nanocrystals in alkaline medium [56,57].

In this regard, the aim of the present work was to synthesize and select the best nanocomposite based on rare earth metal oxide nanoparticles and polysaccharide (dextran-stabilized nanocrystalline cerium oxide) for the subsequent creation of a medical and/or veterinary nanodrug for wound regeneration with a new type of antimicrobial effect.

## 2. Results

### 2.1. Results of Evaluation of Physicochemical Properties of the Synthesized Dextran-CeO_2_ Nanocomposites

**Figure 1:**
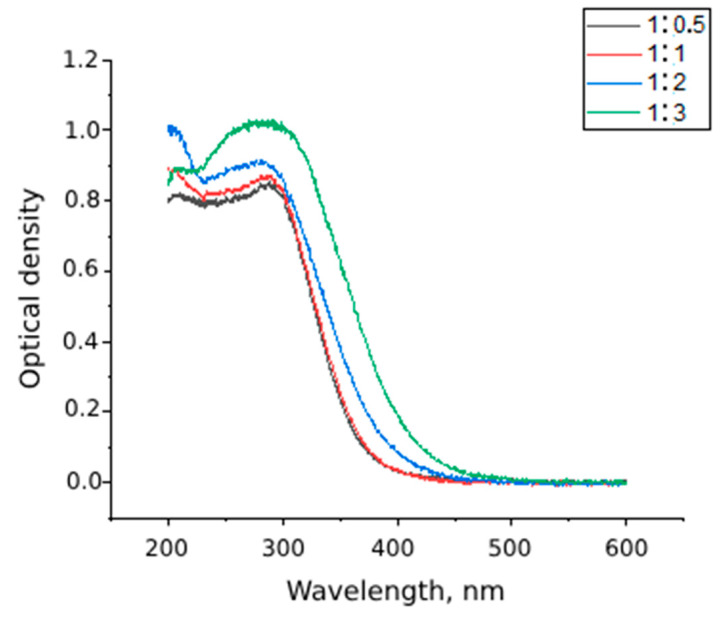
UV-visible spectra of cerium dioxide sols with different cerium: dextran ratios (1:0.5; 1:1; 1:2 and 1:3 by mass).

The results of UV-visible spectroscopy for dextran-coated cerium nanoceria sols indicate the formation of cerium dioxide phase (Figure 1); with increasing dextran content, the absorption band of cerium dioxide shifts to the visible region (up to 400 nm), which indirectly indicates the change in particle size.

**Figure 2:**
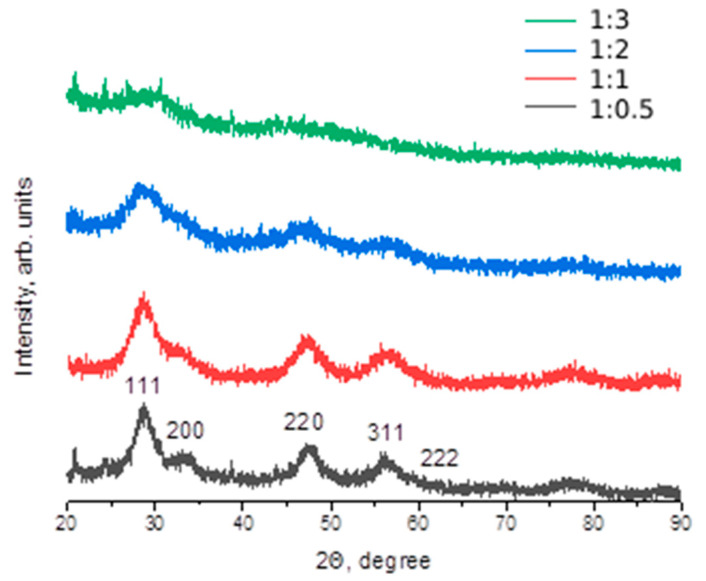
X-ray phase analysis data of dextran-coated nanocrystalline cerium dioxide samples, in different initial product ratios cerium nitrate: dextran (1:0.5; 1:1; 1:2; 1:3 by mass). The crystallite size was estimated using Scherrer formula.

According to the data of X-ray phase analysis, the synthesized variants of nanocomposites are CeO_2_ nanoparticles with a size of 0.5–4 nm (Figure 2). Moreover, the more dextran in the composition of the composite, the less clear the diffraction peaks characteristic of cerium dioxide. This is due to the fact that with increasing concentration of polysaccharide in the composite, the thickness of the dextran coating of nanoceria increases.

**Figure 3:**
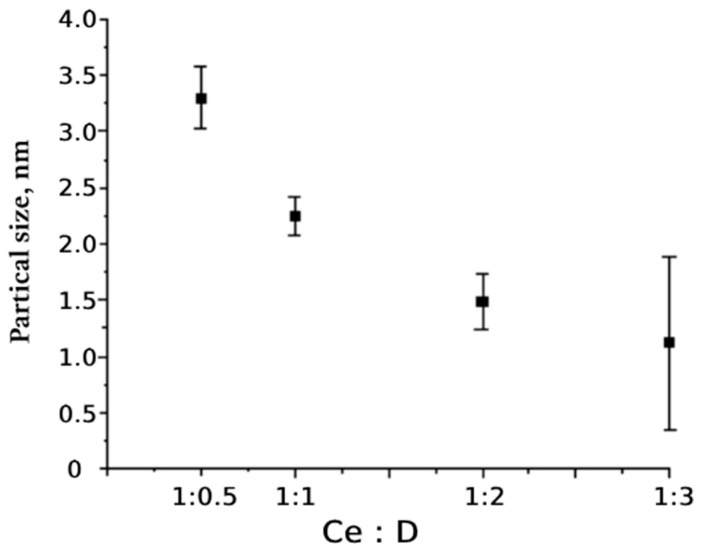
Cerium dioxide particle size obtained in different initial product ratios cerium nitrate: dextran (1:0.5; 1:1; 1:2; 1:3 by mass).

The data on the size of cerium oxide particles obtained from the analysis of diffraction maxima are shown in Figure 3, which shows that the size of cerium nanoparticles decreased as the dextran concentration increased. The largest size of cerium oxide nanocrystals (3.3 nm on average) was recorded at the lowest addition of dextran (Ce0.5D); in the Ce1D (1:1) sample, the size of the nanoparticles was 45% smaller, averaging 2.2 nm. The smallest nanoparticle size was in the Ce2D and Ce3D samples (more than two times smaller than at the minimum dextran concentration). At the same time, the median range of values for the Ce2D composite, bounded by the 25–75% percentiles, was significantly lower (1.2–1.7 nm, Me = 1.5 nm) than for the Ce3D sample (0.4–2.0 nm, Me = 1.2 nm). The data obtained suggest that the strength of the biological effect is greater in Ce2D and Ce3D, which had the smallest nanoparticle core size, of which the Ce2D nanocomposite with minimal variation in nanocrystal size is the most predictable in terms of stability of biological effects.

**Figure 4:**
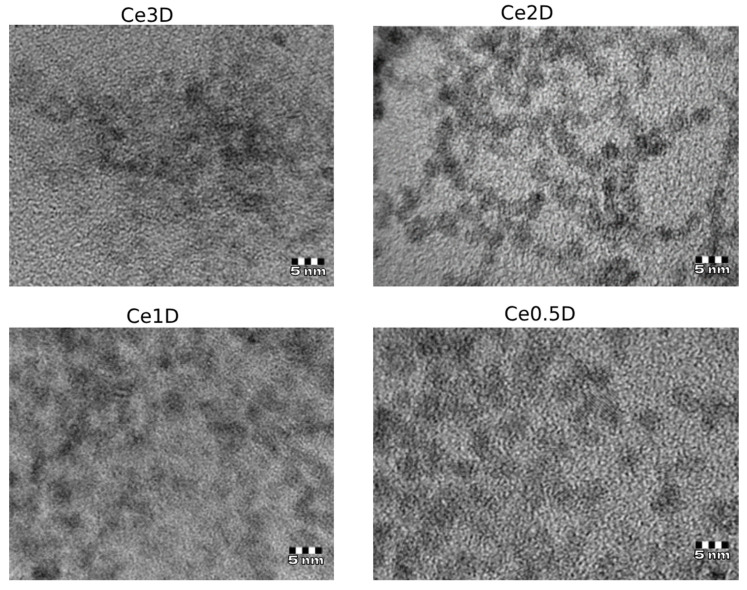
TEM microphotographs of nanocomposites obtained with different dextran content.

Transmission electron microscopy results confirmed that the nanoparticle sizes were less than 5 nm (Figure 4), while increasing the dextran content led to a decrease in particle size to 0.5–2.5 nm in Ce2D and Ce3D, which agrees well with the XRD data.

**Figure 5:**
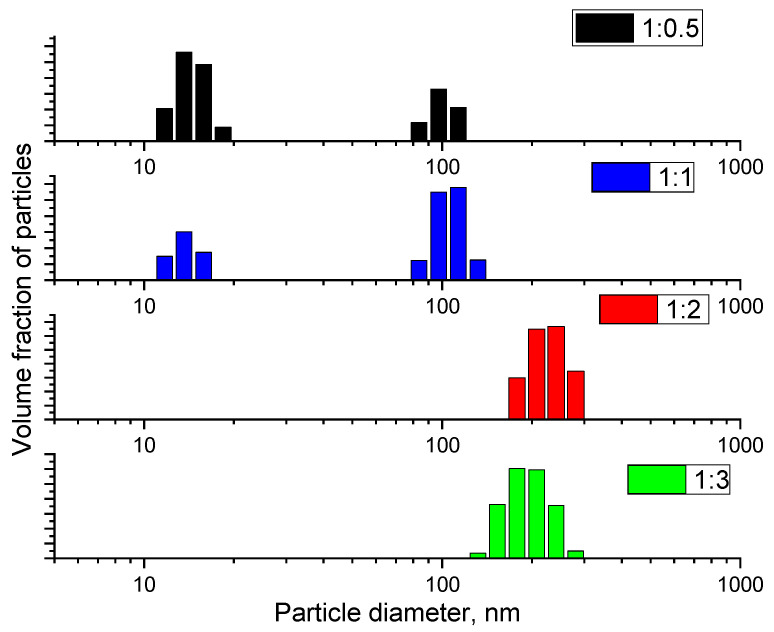
Dynamic light scattering data for cerium dioxide samples stabilized with dextran at different concentrations (1:0.5, 1:1, 1:2, and 1:3).

The results of dynamic light scattering have established that as the content of dextran in the composition of the colloidal solution of cerium dioxide increases, the hydrodynamic radius of the particles increases (Figure 5). This is due to the increase in the number of adsorbed dextran molecules on the surface of cerium dioxide particles; however, it is known that according to X-ray diffraction analysis and electron microscopy data, the size of cerium dioxide particles decreases.

The DLS method allows us to evaluate the aggregative stability of cerium dioxide sols stabilized by dextran without directly estimating the actual size of nanoparticles. The DLS results demonstrate that in the synthesis of cerium dioxide sols, increasing the polysaccharide content from 0.5 to 1 (by mass) leads to the formation of particles with hydrodynamic diameters ranging from 10 to 20 nm and 100 nm; further increasing the dextran concentration to 2 and 3 (by mass) leads to an increase in the diameter of nanocomposites from 110 nm to 200–300 nm.

**Figure 6:**
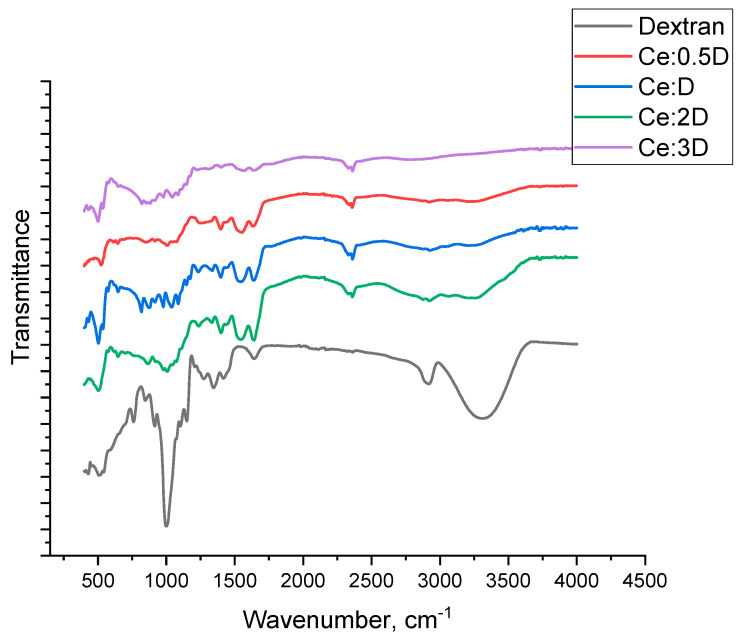
Infrared spectra for cerium dioxide composites stabilized with dextran in different ratios (0.5 to 3).

According to the IR spectroscopy data, all obtained samples are composites of cerium dioxide + dextran composition. Figure 6 shows that the peaks at 3415, 2925, 1648 cm^−1^ characteristic for dextran in other samples of the compositions Ce:0.5D, Ce:1D, Ce:2D, and Ce:3D are absent, which may indicate the formation of a composite of a new composition. One of the main peaks is a strong band in the 3415 cm^−1^ region, which corresponds to an asymmetric O-H vibration that overlaps with hydrogen intramolecular bonding signals. One peak between 2925 and 2932 cm^−1^ can be attributed to the symmetric and asymmetric C-H bonding. The peak at 1648 cm^−1^ corresponds to the aqueous solvate layer of the polysaccharide. Similar peaks are characteristic of a large number of complex polysaccharides such as chitosans, galactans, and glucans. The peak at 1156 cm^−1^ corresponds to the asymmetric stretching of C-O-C and C-C bonds; the peaks around 915 and 845 cm^−1^ indicate the presence of glycosidic bonds.

The above-described peaks on the curves of composites Ce:0.5D, Ce:1D, Ce:2D, and Ce:3D are changed (present with low intensity or absent). This may additionally indicate the formation of new dextran–nanoceria bonds, which have new properties that are not characteristic of dextran. And the available wavelength differences among the nanocomposites indicate different properties of the synthesized nanomaterials differing in the cerium to dextran ratio. Consequently, each nanocomposite may exhibit different biological properties.

### 2.2. Results of Evaluation of the Effect of Nanocomposites on Cytotoxicity, Metabolic and Proliferative Activity of Human Fibroblast Cell Culture

Firstly, the study of the effect of nanocomposites on the metabolic activity of human cells was performed. This is important to study on fibroblast cell cultures in particular, as these cells can be stimulated either in the form of proliferation with an increase in cell population or in the form of increased production of the intercellular substance they produce, which is very important for wound healing in the skin.

**Figure 7:**
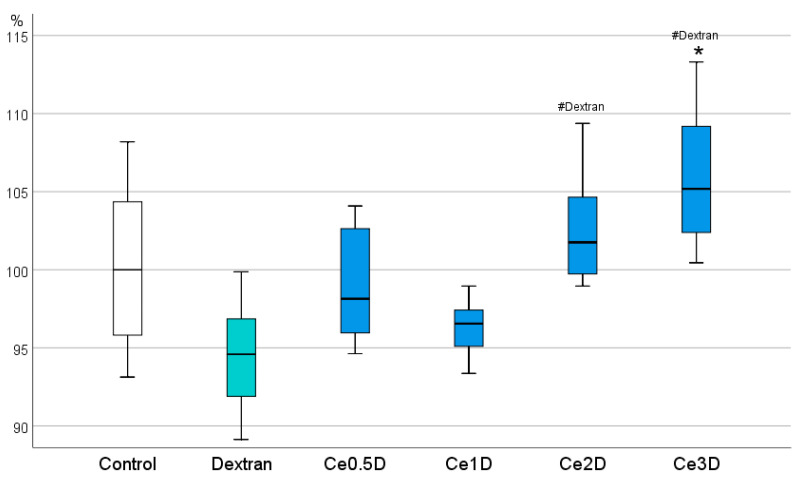
Effect of different concentrations of polysaccharide in cerium oxide nanocomposite + dextran on metabolic activity of human fibroblasts in MTT test, percent of control (ANOVA OD:F = 15,162; df 5, *p* < 0.001; *—different from control at *p* < 0.001; Dunnett t-tests; # dextran-different from dextran comparison group (without nanoceria) at *p* < 0.01, post hoc Bonferroni test).

In the course of analyzing the results of the MTT test, it was found that the metabolic activity of fibroblasts at 72 h of co-cultivation increased with increasing dextran content in the nano-composites studied at a concentration of 10^−2^ M. The highest level, significantly different from both the control group and the comparison group of dextran, was recorded in the Ce3D sols. Moreover, the Ce2D and Ce3D groups did not differ from each other, and in most cases, fibroblast metabolism was higher than in the control (Figure 7). No statistically significant differences were found between Ce0.5D and Ce1D groups. These groups also did not differ from the control and dextran groups, although they were slightly superior to dextran in terms of effect.

The data obtained suggest that the stimulation of fibroblast metabolism probably depends on the concentration of polysaccharide and the size of nanoparticles in the nanocomposite; the smaller the size of nanoparticles of the inorganic core of the nanocomposite (cerium dioxide) and the larger the hydrodynamic polysaccharide radius, the higher the degree of stimulation of metabolic activity of fibroblasts with their production of interstitial wound-filling substance.

**Figure 8:**
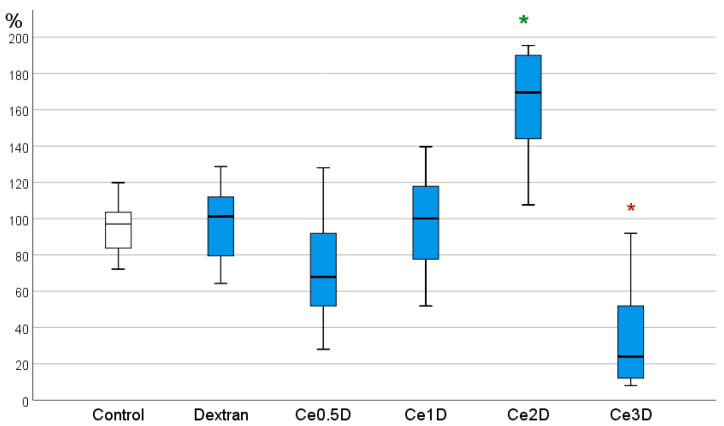
Effect of nanocomposites based on polysaccharide dextran and cerium oxide nanoparticles at concentrations of 10^−2^ M on the proliferative activity of fibroblasts (BJTERT cell line) by direct cell counting using an automated cell counter. Mean percentages from control are presented (ANOVA OD:F = 20.781; df 5, *p* < 0.001; difference from control at *—*p* < 0.001 Bonferroni and Dunnett *t*-tests). Green *—stimulation, red—suppression of proliferation.

Cell counting after 72 h of co-culture determined inter-group differences, indicating the effect of nanocomposites at a concentration of 10^−2^ M on human fibroblast proliferation. A clear advantage of the Ce2D nanocomposite was demonstrated, where a 107–291% stimulation of cell division was recorded, with an average of 172.1% ± 46.8% relative to control (*p* < 0.001). In the groups of dextran, Ce0.5D, and Ce1D, no statistically significant differences in cell number were registered relative to the control, while in the Ce0.5D group, an unreliable tendency to inhibition of fibroblast proliferation by an average of 23% relative to the control was determined. Ce3D nanocomposite at a concentration of 10^−3^ M was found to significantly inhibit fibroblast proliferation by an average of 75% relative to control (34.6 ± 27.4%, *p* < 0.001). According to ANOVA Bonferroni post hoc test, statistically significant differences were recorded between the performance of Ce2D and Ce3D nanocomposites from all other studied groups. Ce2D was the best in this parameter, while Ce3D was the worst (Figure 8).

The obtained results prompted us to perform cell counting at different concentrations of all studied nanocomposites. For this purpose, sols at concentrations of 10^−3^ M, 10^−4^ M, 10^−5^ M, and 10^−6^ M were prepared using water for injection.

**Figure 9:**
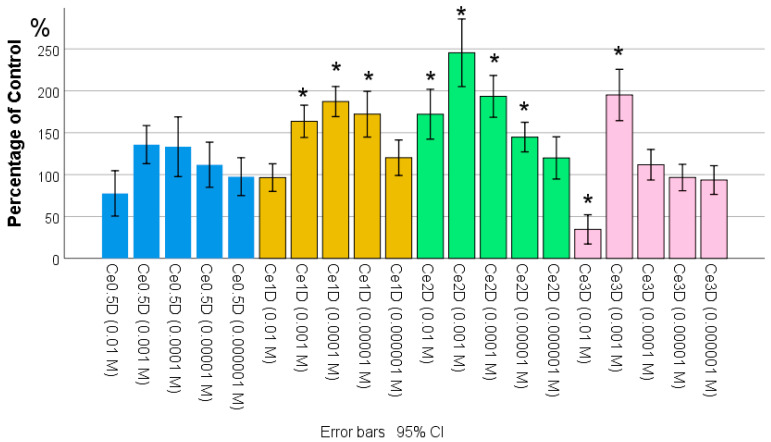
Effect of nanocomposites based on cerium oxide polysaccharide dextran polysaccharide in a wide concentration range of 10^−2^ M–10^−6^ M on the proliferative activity of human fibroblasts by direct cell counting. Mean percentages from control are presented (ANOVA OD:F = 19.703; df 20, *p* < 0.001; *—difference significant from control at *—*p* < 0.05 Dunnett *t*-tests).

From the results of direct cell counting, it was determined that fibroblast proliferation was enhanced to the greatest extent and over the widest range of concentrations when co-cultured with the Ce2D nanocomposite (172% on average at a concentration of 10^−2^ M, 245% at 10^−3^ M concentration, 193% at 10^−4^ M concentration, and by 145% relative to control at 10^−5^ M concentration, *p* < 0.05), with the best effect, significantly superior to all other subgroups, registered when exposed to Ce2D at 10^−3^ M concentration. On average, the number of fibroblasts after 72 h of co-cultivation in the Ce2D sample at 10^−3^ M concentration was 245 ± 63% relative to the control. The Ce0.5D nanocomposite had no effect on cell numbers at all concentrations. The Ce1D nanocomposite enhanced proliferation of human fibroblasts at concentrations of 10^−3^ to 10^−5^ M by an average of 164–187% relative to control (*p* < 0.05). Ce3D significantly enhanced fibroblast proliferation only at concentrations of 10^−3^ M by an average of 196% (Figure 9).

It is interesting to note the fact that the maximum efficiency of all nanocomposites was registered at the concentration of 10^−3^ M with a clear advantage of Ce2D composite, at which the number of fibroblasts after 72 h was 2.5 times higher than in the control (at the concentration of 10^−3^ M Ce0.5D—1.4 times, Ce1D—1.5 times, Ce3D—2.0 times relative to the control, *p* < 0.01).

**Figure 10:**
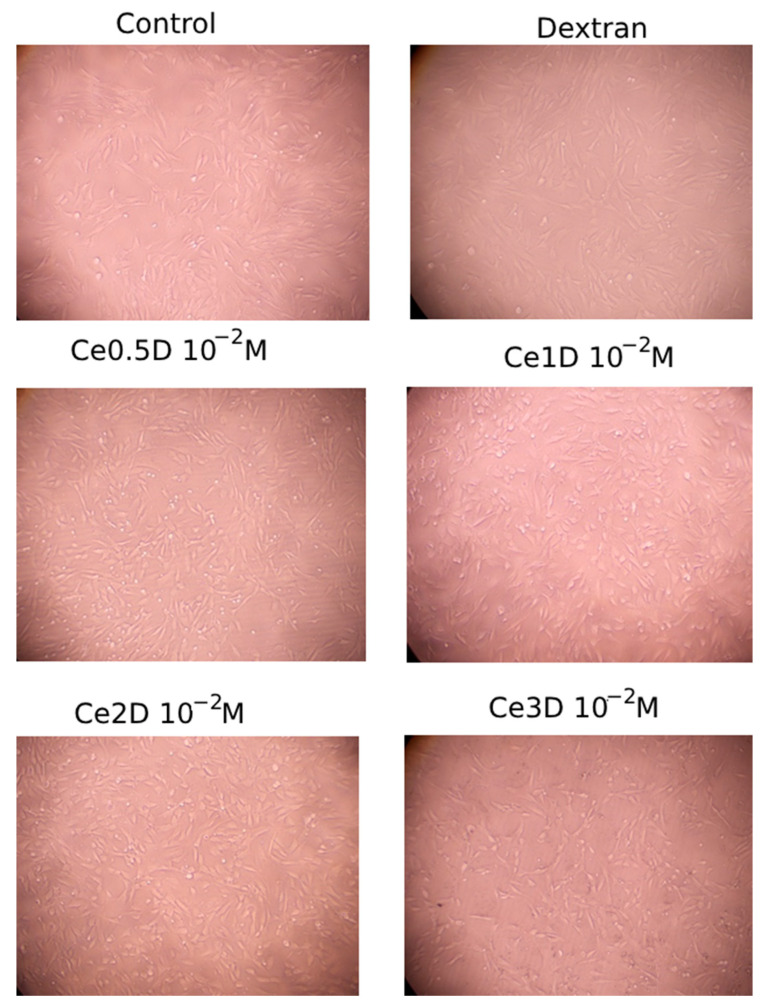
Photographs of fibroblasts after 72 h of co-cultivation with nanocomposites (×10 magnification).

To determine the cytotoxicity and safety of the synthesized nanocomposites, cells were visualized by light microscopy (Figure 10) and the percentage of dead cells was evaluated. At a high concentration (10^−2^ M of cerium dioxide), 100% cell survival in all 12 samples was recorded in the control, Ce2D, and Ce3D groups. The latter situation (absence of cell death along with their normal visualization) against the background of cell division inhibition proves the safety of the samples, with living cells spending energy not for proliferation but for collagen and elastin synthesis, which is confirmed by MTT-test data.

The most frequent dead cells were determined in the Ce0.5D group (16.7% of cases registered 2–29% dead cells). Single cases of dead cells were reported in the dextran and Ce1D groups (dextran: 25% of cases (3 out of 12 cells) reported 2% to 5% dead cells; Ce1D: 16.7% of cases (2 out of 12 cells) reported 5% dead cells). Although this is not an indication of cytotoxicity (acceptable values), it is reasonable to select Ce2D and Ce3D for future drug development.

At other concentrations, more dilute sols also showed advantages of Ce2D and Ce3D nanocomposites, with 100% survival (no dead cells) for both nanocomposites with excess dextran recorded at concentrations of 10^−2^ M, 10^−3^ M and 10^−6^ M (otherwise up to 2–5% dead cells in 1–2 out of 12 samples). In the samples with Ce1D nanocomposite, insignificant amounts (up to 5%) of dead cells in 1–2 out of 12 wells were recorded at all concentrations. In samples with the Ce0.5D nanocomposite, there were no dead cells only at the lowest concentrations (10^−5^–10^−6^ M).

Thus, the most preferred and safe nanocomposite for the future development of a drug to accelerate wound healing is Ce2D, characterized by a balance of simultaneous stimulation of both cell proliferation and metabolism with the most predictable dose-dependent effect.

### 2.3. Antimicrobial Activity of Nanocomposites

The study by gas chromatography with mass spectrometry revealed that cerium oxide-based nanocomposites coated with dextran in different ratios affect *E. coli* abundance with a significant bacteriostatic effect at all cerium to dextran ratios after 48 h of incubation, but after 24 h of incubation, this only occurred with two types of composites (Ce0.5D and Ce2D).

**Figure 11:**
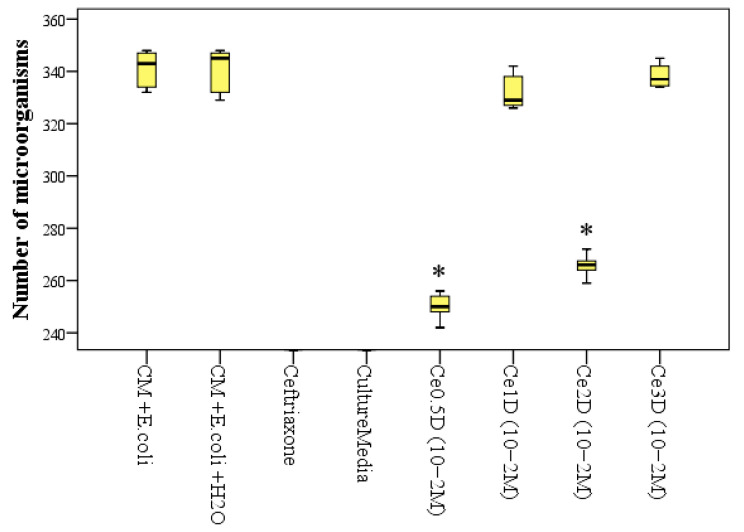
Number of *E. coli* microbial bodies (×10^5^/g) in control groups and when co-cultured with sols of dextran-coated nanoceria in different cerium–dextran ratios, after 24 h of incubation (*—significant difference from control (culture media and *E. coli*) at *p* < 0.01; ANOVA test including posterior Dunnet tests).

Thus, after 24 h of incubation in the control groups, where there was only nutrient medium and *E. coli*, the average number of microbial bodies was on average 1.36 times higher than in samples with the addition of 10 vol% Ce0.5D at a dose of 10^−2^ M, where the number of microbial bodies averaged 250 ± 5.5 × 10^5^ cells/g medium (*p* < 0.01) and 1.28 times higher than in samples with the addition of Ce2D at the same dose of 10^−2^ M (*p* < 0.01), where the number of *E. coli* averaged 265.7 ± 4.1 × 10^5^ cells/g medium. Accordingly, the percentage of significant suppression of *E. coli* growth when co-cultured with nanocomposites after 24 h was 26.7% when incubated with Ce0.5D and 22.1% when incubated with Ce2D (*p* < 0.01). There was no statistically significant difference in this index between the Ce0.5D and Ce2D groups (*p* = 0.071 with Bonferroni correction for multiple comparisons). At the same time, both groups Ce0.5D and Ce2D were significantly different from groups Ce1D and Ce3D, in which the number of microbial bodies after 24 h was 25–34% higher and averaged 332.4 ± 7.2 and 335.2 ± 8.1 × 10^5^ cells/g medium, respectively; this did not differ from the control groups (*p* > 0.05) (Figure 11).

An ANOVA test determined that multiple differences were significant after 24 h (F = 2805.9; *p* < 0.001). However, after 48 h, all types of nanocomposites at a concentration of 10^−2^ M ceria showed significant antimicrobial (bacteriostatic) activity compared to the control groups.

Thus, after 48 h of incubation in the thermostat, the values of the number of microbial cells (×10^5^/g) were as follows: in the Ce0.5D group—609.4 ± 9.7; Ce1D—796.8 ± 9.9; Ce2D—653.8 ± 6.9; Ce3D—1098.2 ± 18.9 cells per gram of medium. These results were statistically significantly different from those of the control groups. In the control tubes, the average number of microorganisms was 4.3 times higher than in the Ce0.5D group (*p* < 0.01), 3.3 times higher than in the Ce1D group (*p* < 0.01), 4.0 times higher than in the Ce2D group (*p* < 0.01), and 2.4 times higher than in the Ce3D group (*p* < 0.01). Consequently, the application of cerium–dextran nanocomposites at a concentration of 10^−2^ M, can inhibit the growth and multiplication of *E. coli* by an average of 77% with Ce0.5D, 70% with Ce1D, 75% with Ce2D and 58% with Ce3D.

**Figure 12:**
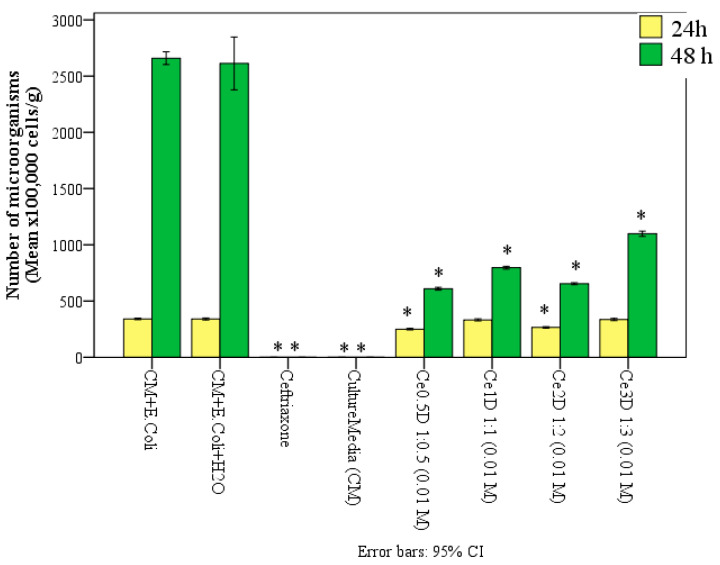
Number of *E. coli* microbial cells (×10^5^/g) in control groups and when co-cultured with 10 vol.% sols of nanocomposites with different dextran concentration, after 24 h and 48 h (*—significant difference from control (culture media and *E. coli*) at *p* < 0.01; ANOVA including Dunnett’s posterior tests).

After 48 h, according to GC-MS data, the number of *E. coli* in the control groups significantly increased on average by 7.7 times, while during incubation with nanocomposites, this increased by 2.4–3.3 times. Namely, this increased by 2.44 times with Ce0.5D, 2.40 times with Ce1D, 2.46 times with Ce2D, and 3.29 times with Ce3D. Thus, at a ratio of 0.5–2 of dextran in the synthesis of nano-ceria oxide, the number of *E. coli* increased equally on average by 2.4 times, which is significantly less than in the control groups, i.e., at these ratios of cerium/dextran (Ce0.5D, Ce1D and Ce2D), the multiplication of *E. coli* was inhibited equally on average by 3.2 times compared to the control groups (*p* < 0.01). Less pronounced bacteriostatic activity was possessed by Ce3D, in which the number of microorganisms for the second day of the study increased by 3.29 times, although a significant antimicrobial effect was established in the form of inhibition of *E. coli* colony growth relative to the control by 2.3 times (*p* < 0.01) (Figure 12). The ANOVA test established the significance of multiple differences after 48 h of co-culture of dextran nano-composites (F = 448.7; *p* < 0.001).

Thus, the best antimicrobial activity in the form of bacteriostatic effect against Gram-negative bacterium *E. coli* was found in Ce0.5D and Ce2D nanocomposites.

Since the Ce2D nanocomposite was selected according to previous studies, the next stage of microbiological study was to determine the antimicrobial activity of this nanocomposite, but at different concentrations (10^−2^–10^−5^ M).

In the results of gas chromatography with mass spectrometry after 24 h of incubation of Ce2D with nutrient medium containing *E. coli*, only at a concentration of 10^−2^ M was a significant antimicrobial effect established, confirming the results of the previous experiment in the form of a reduction in the number of microbial bodies (1.3 times more microbes in the control; *p* < 0.01). At other concentrations of Ce2D, no bacteriostatic effect was detected after 24 h, and the results were comparable with the control groups.

**Figure 13:**
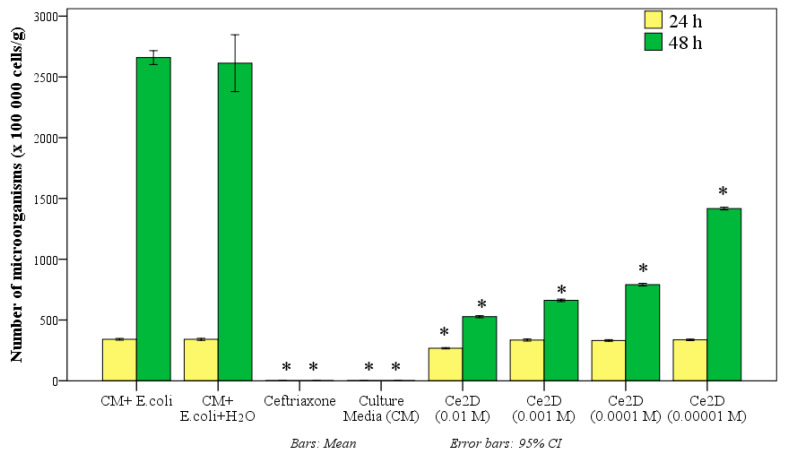
Number of *E. coli* microbial cells (×10^5^/g medium) in control groups and when co-cultured with 10 vol.% Ce2D nanocomposite sols at different concentrations after 24 h and 48 h (*—significant difference from control (culture media and *E. coli*) at *p* < 0.01; ANOVA including Dunnett’s posterior tests).

After 48 h, a bacteriostatic effect against *E. coli* was established for all concentrations of Ce2D. The values of microbial cell counts (×10^5^/g) in Ce2D groups averaged 1417 ± 13.8 ×10^5^ at 10^−5^ M concentration; 791 ± 12.9 at 10^−4^M concentration; 661.5 ± 12.9 at 10^−3^ M concentration; and 526.9 ± 10.7 ×10^5^ cells per gram of medium at 10^−2^ M concentration. These statistically significantly differed from the control groups by an average of 1.8 times, 3.3 times, 4.0 times, and 5.0 times, respectively (*p* < 0.01) (Figure 13). Consequently, the application of Ce2D is able to inhibit *E. coli* multiplication by 47–80% (at a concentration of 10^−5^ M by 47% on average, 10^−4^ M by 70%, 10^−3^ M by 75%, and 10^−2^ M by up to 80%). ANOVA analysis established the significance of multiple differences after 24 h (F = 2276.3; *p* < 0.001) and after 48 h (F = 638.8; *p* < 0.001).

A significant dose-dependent effect of dextran nanoceria on *E. coli* multiplication was established. In the group with the lowest concentration of nano-ceria (10^−5^ M), the number of microbial bodies was significantly higher than in all other groups (on average 1.8 times higher than at 10^−4^ M (*p* < 0.01), 2.1 times higher than at 10^−3^ M (*p* < 0.01), and 2.7 times higher than at 10^−2^ M (*p* < 0.01). The number of microbial bodies was significantly lower in the group with the highest concentration of nano-ceria than in the groups with concentrations of 10^−4^ M and 10^−5^ M (*p* < 0.01). The group with 10^−3^ M concentration of Ce2D was not statistically different from the group with 10^−2^ M concentration (*p* > 0.05); that is, the antimicrobial activity of 10^−2^–10^−3^ M after 48 h is comparable.

If in control groups, the number of microorganisms for a day (from 24h to 48h) increased on average by 7.8 times, then during incubation with Ce2D at concentrations of 10^−2^ M and 10^−3^ M, the number of *E. coli* increased only by 2.0 times, at a concentration of 10^−4^ M—on average by 2.4 times, and at a concentration of 10^−4^ M—on average by 4.2 times. Consequently, with the application of Ce2D composite at concentration of 10^−2^ to 10^−3^ M, it was possible to reduce the reproduction of microorganisms within 2 days by 3.9 times, at a concentration of 10^−4^ M—3.2 times, and at a concentration of 10^−5^ M—1.8 times.

Thus, it was proved that after 24 h, nanocomposite Ce2D in the maximum concentration of 10^−2^ M can significantly inhibit the multiplication of *E. coli* on average by 22%. After 48h, all concentrations of Ce2D showed bacteriostatic activity, inhibiting *E. coli* multiplication by 47–80%, and a dose-dependent effect was established. The higher the concentration of nano-Ce2D, the greater the antimicrobial (bacteriostatic) activity. After 48 h, the number of microbial bodies at a dose of 10^−3^ M Ce2D is comparable to the concentration of 10^−2^ M. The application of Ce2D nanocomposite at a dose of 10^−2^–10^−3^ M allows a reduction in the rate of microorganism multiplication in the period from 24h to 48h by 3.9 times (at the dose of 10^−4^ M—3.2 times, at 10^−5^ M—1.8 times).

All this makes the Ce2D nanocomposite at a concentration of 10^−2^–10^−3^ M attractive for the further development of a medical drug with a new type of antimicrobial activity.

## 3. Materials and Methods

### 3.1. Synthesis of Nanocomposites Using Dextran and Cerium Nitrate in Different Ratios

We used a modified methodology published in our earlier papers [35,39]. Cerium oxide nanoparticles coated with the polysaccharide dextran were synthesized by the following methods. First, a 50 mL mixed solution (distilled water) was prepared consisting of Cerium (III) nitrate hexohydrate (Ce(NO_3_)_3_x6H_2_O) (99.99%, LANHIT, Russia, Moscow), molecular weight 434.23 (326.13 anhydrous) and Dextran Mr = 6000 Da (ABCR GmbH, Karlsruhe, Germany) in four different Ce(NO_3_)_3_x6H_2_O: dextran ratios (by weight): (1) 1:0.5 (Ce0.5D); (2) 1:1 (Ce1D); (3) 1:2 (Ce2D); (4) 1:3 (Ce3D). That is, 1.0 g of cerium(III) nitrate and 0.5 g of dextran were used for the 1st variant; 2nd variant—1.0 g of cerium(III) nitrate and 1.0 g of dextran; 3rd variant—1.0 g of cerium(III) nitrate and 2.0 g of dextran; 4th variant—1.0 g of cerium(III) nitrate and 3.0 g of dextran. In all compositions, the concentration of the initial active substance cerium nitrate was the same everywhere during each synthesis; only the amount of dextran changed.

The solution was continuously stirred on a magnetic stirrer, to which 3 M aqueous ammonia solution (special purity, Himmed, Moscow, Russia) was added dropwise for 3 h, maintaining pH at 7.5–8.0. The pH solutions were measured using a Crison GLP 22 pH-meter (Crison Instruments, SA, Barcelona, Spain) equipped with a Crison 5201 combination electrode and providing an accuracy of ±0.003 pH in the range from 0 °C to 100 °C.

When the pH became constant, the mixture was additionally stirred for 2 h. Then, aqueous ammonia solution was added up to pH = 12 followed by additional stirring for at least 8 h. The end of formation of cerium dioxide particles during the experiment was monitored by UV-visible absorption spectra. The experiment was considered complete when the peak characteristic of Ce^3+^ disappeared in the spectra. The complete oxidation was ensured by differential UV-vis spectroscopy by disappearance of local minimum on differential spectra at 280–290 nm.

For cerium dioxide samples with different dextran contents, the time to complete the synthesis was different. The longest synthesis was required for the nanocomposite with the highest dextran content (48 h).

Then, excess isopropanol (350 mL) was added to the obtained solution until a yellow precipitate was formed. The precipitate was further washed several times with isopropanol (Chimmed, Moscow, Russia), centrifuged at ~18,000 RCF (relative centrifugal force) and dried at 50 °C. Dextran-stabilized cerium sol was obtained by dispersing the powder in distilled water. 

Then, thermogravimetry was performed, based on the results of which the concentration was calculated and solutions with different concentrations were prepared for biological studies. We used alund crucibles, brought them to constant mass, weighed them, placed aliquots of the sol inside, dried and annealed them at 900 °C, and weighed again. The weight form was considered to be CeO_2_. From the mass difference, the mass of cerium oxide was determined and converted to the concentration of the sol.

To determine the stability of the hydrosols, the hydrosols were observed for at least 3 months after their synthesis, keeping the samples under normal conditions at room temperature. The obtained nanoceria sols stabilized with dextran did not require additional stabilization, and after 1, 2 and 3 months, they had a stable pH value (from 6.8 to 7.4), were easily diluted with solutions and did not lose their aggregative stability (transparent solution of yellowish color did not change, no precipitate). After stability evaluation, the nanocomposite sols were investigated in biological (cellular and microbiological) experiments.

### 3.2. Studied Samples and Comparison Groups

Depending on the initial ratio of cerium nitrate to dextran, in this work, we evaluated 4 types of nanoceria sols at a concentration of 10^−2^ M: Ce0.5D (at a ratio of 1:0.5), Ce1D (at a ratio of 1:1), Ce2D (at a ratio of 1:2) and Ce3D (at a ratio of 1:3). Also, in biomedical experiments, sterile water for injection was used to study the effects of different concentrations of the obtained sols, and concentrations of 10^−2^ M, 10^−3^ M, 10^−4^ M, 10^−5^ M and 10^−6^ M were obtained.

Nanoparticle concentrations are expressed as M (mol/L). This is the molarity per formula unit of CeO_2_, i.e., actually a mole of cerium dioxide per liter of colloidal solution. 

The control was the water with which the dilution was performed, used in the same volume as the sols. In addition, the results obtained were compared with a group of dextran (Mr = 6000, ABCR GmbH, Karlsruhe, Germany) used to prepare the sols. Therefore, the comparison group was dextran without cerium (0.3 g of dextran was dissolved in 50 mL of distilled water).

Microbiological experiments were also controlled by groups with antibiotic (ceftriaxone).

### 3.3. Methods of Evaluation of Physicochemical Properties of Nanocomposites

The obtained samples of nanomaterials were characterized by UV-visible spectroscopy, transmission electron microscopy, X-ray phase analysis, dynamic light scattering (DLS), and infrared spectroscopy.

Ultraviolet–visible spectroscopy was performed on a spectrophotometer SF-2000 (OKB Spektr, Saint Petersburg, Russia), working on a single-beam scheme. Imaging was performed in the wavelength range from 190 to 800 nm with a step of 0.1 nm, and the optical slit width was 0.2 nm. Imaging in the range from 190 to 394.9 nm was performed using a deuterium lamp, and from 395 to 800 nm using a halogen lamp. The exposure time was 50 msec. Each spectrum was imaged 10 times with subsequent averaging of the results.

Transmission electron microscopy (TEM) of the synthesized nanocomposites was carried out on a JEM 2100 JEOL electron microscope (JEOL Ltd., Tokyo, Japan) with an accelerating voltage of 200 kV.

X-ray phase analysis of samples was carried out on a Rigaku D/MAX 2500 diffractometer (CuK-radiation) at a goniometer rotation speed of 1–2 °2θ/min (Rigaku Corporation, Tokyo, Japan). The identification of diffraction maxima was carried out using the International Center for Diffraction Data (Joint Committee on Powder Diffraction Standards (JCPDS) data bank, PA, USA).

The sols were studied by dynamic light scattering and zeta potential measurements at 20 °C using a Photocor Compact-Z analyzer (Photocor LLC, Moscow, Russia). The correlation function for each sample was obtained by averaging 10 curves (the accumulation time of one curve was 20 s). The hydrodynamic diameter of particles was determined using the regularization method (DynalS software, available online: http://www.softscientific.com/science/WhitePapers/dynals1/dynals100.htm, accessed on 17 May 2024).

Dynamic light scattering (DLS) studies were performed using a Zetasizer Nano ZS laser analyzer with a 633 nm laser (Malvern Instruments Limited, Malvern, Worcestershire, UK).

Infrared spectroscopy analysis of nanoceria–dextran composites was performed on a Perkin Elmer Spectrum 65 FTIR spectrometer (PerkinElmer, Waltham, MA, USA).

### 3.4. Methods of Evaluation of the Effect of Cerium–Dextran Sols on Cytotoxicity, Metabolic and Proliferative Activity of Human Fibroblast Cell Culture

The study was performed on human fibroblast culture (BJTERT line) derived from neonatal foreskin. The origin of the line is the ATCC collection of typed cultures (Manassas, VA, USA).

#### 3.4.1. Cell Culturing

Human immortalized fibroblasts of BJ TERT line were cultured in DMEM (Dulbecco’s Modified Eagle’s Medium) (Paneco, Moscow, Russia) supplemented with 10% fetal calf serum (Global Kang Biotechnology, Qinhuangdao, China), 1% penicillin/streptomycin, and 0.32 mg/mL glutamine (Paneco, Russia). Cell passaging was performed every 7 days according to a standard protocol, and the medium was changed every 3 days between passages. Cells were cultured in a CO_2_ incubator (Binder, Tuttlingen, Germany), and incubation temperature was 37 °C in a humid atmosphere with 5% carbon dioxide in the air.

For the experiment, human immortalized fibroblasts of BJ TERT line were seeded in 24-well plates (NEST, Wuxi, China) at a cell concentration of 5 × 10^4^ cells/mL in suspension. After 24 h, the test substances were added at concentrations according to the experiment design in a volume of 100 μL. Incubation was then continued under standard controlled CO_2_ incubator conditions for 72 h. Equivalent volumes of 0.9% sodium chloride solution were added as a control. At the end of the incubation time, proliferative activity evaluation tests were performed.

#### 3.4.2. MTT Test

An MTT assay was used to determine metabolic activity and to assess changes in proliferative activity.

The MTT assay was performed according to a standard protocol, according to which MTT salt (3-(4,5-dimethylthiazol-2-yl)-2,5-diphenyltetrazolium bromide, Neofroxx, Germany) was dissolved in PBS (stock solution; 5 mg/mL). Next, a working solution of MTT (0.5 mg/mL) was prepared by dissolving the stock solution in the culture medium. Then, the medium containing nano-ceria oxide compounds was removed from the culture plates, and the MTT working solution was added to each well for 30 min at 37 °C. After the MTT solution was removed, DMSO (PanReac AppliChem, Darmstadt, Germany) was added for 5 min at room temperature on an Elmi-S4 oscillating shaker (ELMI Ltd., Riga, Latvia). Finally, the solution was transferred to a 96-well plate, and the absorbance was recorded on a spectrophotometer (Multiscan, Labsystems, Vantaa, Finland) set at λ = 540 nm. The final measurement result was expressed in relative optical density (OD) units.

#### 3.4.3. Determination of Fibroblast Proliferative Activity and Assessment of Cytotoxicity by Cell Counting and Assessment of Plasma Membrane Integrity by Trypan Blue Staining

The cultivation of immortalized human fibroblasts was performed according to the protocol described above. At the end of incubation with nanocomposites, cells were detached and counted automatically using Countess II Automated Cell Counter (Thermo Scientific, Waltham, USA) in special plastic disposable slides (RWD, Shenzhen, China) following the manufacturer’s protocol. The procedure allowed, in addition to counting the total number of cells, the determination of cell viability by penetration through the cell membrane and staining of non-viable cells with trypan blue solution [58]. Briefly, after the detachment of cells by trypsinization (trypsin: Versen solution (Paneco, Moscow, Russia) at a ratio of 1:4), 0.4% trypan blue solution (Paneco, Moscow, Russia) was added to the cell suspension. The solution was mixed by pipetting. Then, the stained cell suspension was introduced into a slide, which was placed in a counter for automatic counting. The total number of cells per unit volume (×10^5^ cells/mL) and the percentage of live and dead cells were counted as the result.

When performing cell experiments, each sample was tested at 12 repetitions in both the counter and MTT test to ensure reliable results and valid conclusions.

### 3.5. Determination of Antimicrobial Activity of Cerium–Dextran Nanocomposites by Mass Spectrometry of Microbial Markers Using Gas Chromatograph with Mass-Selective Detector

In the course of our previous studies, we proved that the antimicrobial effect of nanocrystalline cerium oxide sols using classical methods (diffusion into agar, serial dilution method) is not reasonable to study, since nano-cerium does not diffuse into agar and its mechanism of action does not comply with the standard rules of pharmacopoeia [35], which is actually associated with contradictory results of different researchers (there are works claiming that metals nanoparticles have a pronounced antimicrobial effect [33,59,60,61,62,63]; at the same time, there are many works in which the authors did not obtain such an effect [64,65,66]). 

This study was performed on a gas chromatograph with mass-selective detector (GC-MS) “Maestro-aMS” (Interlab, Moscow, Russia). The method is based on the high-precision determination of the presence of molecular features of microorganism-specific markers (higher fatty acids, aldehydes, alcohols and sterols in the sample under study), i.e., the fatty acid status of the microorganism, which is specific and genetically determined. The microbiological analyzer Maestro allows the accurate quantification of the content of microorganisms in any biological sample. Although the automatic algorithm of the device allows the determination of 50 microorganisms simultaneously, in the present study, we investigated the effect of nanoceria on the growth inhibition of one Gram-negative bacterium, *Escherichia coli* (*E. coli* ATCC 8739), using a test strain from the collection of the A.A. Tarasevich Research Institute for Standardization and Control of Medical Biological Preparations (Moscow). The method is characterized by high sensitivity (able to detect 10^3^ cells in the sample). The final calculation of the exact number of microorganisms was expressed as number × 10^5^ microbial cells per gram of tested material.

Two stages of experiments were performed. In the 1st series, a total of 8 groups were studied (Table 1). The studied 4 groups of nanocomposite sols (Ce0.5D, Ce1D, Ce2D, Ce3D) were of one concentration, 10^−2^ M. Control groups: Culture media (CM) represented meat-peptone broth (MPB), CM + *E. coli*, CM + *E. coli* + H_2_O (sterile water for injection in the same volume as the nanocomposite was used). Ceftriaxone III-generation cephalosporin antibiotic (ZAO Rafarma, Russia, 1g powder pack) was chosen as a reference comparison to evaluate the antimicrobial activity, to prove the antibacterial effect against strains of microorganisms. In the experiment, we used a concentration of 100 mg/mL, dissolving ceftriaxone powder (contents of the vial, 1 g) with sterile water for injection in a volume of 9.6 mL.

In each tube of all groups, the initial quantitative content in 0.5 mL of *E. coli* suspension was 5 × 10^5^ cells.

The tubes with the contents were placed in a thermostat at 37 °C for 24 h; then, an aliquot was taken and analyzed by gas chromatography-mass spectrometer (GC-MS), and the remaining tubes were placed in the thermostat for another 24 h to repeat the GC-MS study after 48 h of co-cultivation.

In the 2nd series of experiments, the antimicrobial activity of different concentrations of the selected nanocomposite was investigated using a similar methodology. Eight groups were also studied. The control and comparison groups did not change; only the studied groups changed, of which there were 4, each with different concentrations of the selected nanocomposite (10^−2^ M, 10^−3^ M, 10^−4^ M and 10^−5^ M).

Despite the high accuracy of the device, each sample of all series of experiments was tested at least five times (five repetitions).

### 3.6. Statistical Analysis

For the creation of the graphs and to analyze the data of the nanoceria physicochemical characterization, OriginPro 2018 from OriginLab software SR1 (Northampton, MA, USA) was used.

Statistical processing of the results of biomedical research was carried out using the statistical program SPSS 25.0 (IBM Company, New York, NY, USA). First of all, the normality of the distributions of indicators was assessed using the Kolmogorov–Smirnov and Shapiro–Wilk criteria. All samples and their groups obeyed the law of normal distribution. After that, we performed descriptive statistics of continuous quantitative indicators, which obeyed the law of normal distribution, in the form of mean, std. deviation, std. error, 95% confidence interval for mean (95CI), minimum, and maximum. In the cell experiments, the mean value was determined in the control group, which had only cell medium containing fibroblasts and 100 μL of diluent (the solution with which the nanoceria was diluted to create the specified concentrations). Relative to the mean value in each experiment, the percentages in the studied groups were calculated, obtaining the final figure, the percentage of the control. One-factor ANOVA analysis of variance was performed for comparative analysis of different subgroups of the test. Posterior multiple comparisons were performed using Dunnett’s test (for comparison with controls) and Bonferroni’s test. Differences were considered statistically significant at *p*-value < 0.05.

## 4. Discussion

Currently, there is an active search for optimal ways to create new wound-healing drugs with a better balance of regenerative and antimicrobial effect of a new type that does not cause antibiotic resistance. Great hopes are placed on nanocomposites based on cerium oxide nanoparticles [32,33,34,35,36,37,38,39], which is a rare earth metal with variable valence, coated with a polymer shell, dextran [50,51,52,53,54], which is widely used in medicine [67,68,69]. This complex seems to be an excellent combination for the development of a medical drug that effectively accelerates wound healing.

In order to achieve our aim, we had to solve both chemical–physical and biological problems in the process of experimentation on the preparation of nanocomposite construction from cerium oxide as the core of the particle and dextran as the outer coating of the particle. First, the size dependence of the created composites on the concentration of dextran in the nanoceria sols was confirmed using physicochemical methods. Direct illustrations of the created composites obtained by transmission electron microscopy confirm this dependence while maintaining the relative dimensional structure and size of the cerium nanocrystal particle itself in the required sizes up to 5 nm. However, the size structure of the synthesized nanocomposites turned out to be different. The change in the nanocomposite size, directly proportional to the dextran concentration in the sol, was logically explained by the higher degree of deposition of dextran molecules on the cerium dioxide crystal compared to other groups. An initially lower concentration of cerium nitrate in the system with high concentration of dextran correlates with an increase in the thickness of dextran coatings, which reduced the diffraction peaks characteristic of cerium dioxide and, therefore, changed the degree of contacts, and thus the activity of the nanocomposite itself with respect to biological objects. The higher concentration of dextran in the nanosol led to relatively faster blocking of aggregation (slugging) of nanoceria particles. This statement is confirmed by the analysis of diffraction maxima, which showed an inversely proportional dependence of the nanocomposite core size on the dextran concentration in the nanosol. This means that it is possible, by changing only the dextran concentration or synthesis time, to model nanoparticles of the required size of both the core size and the thickness of the polymer coat.

Infrared spectroscopy demonstrated that the nanocomposite particles obtained in the process of synthesis are indeed composite and consist of cerium dioxide and dextran, and the differences in wavelengths of the synthesized nanocomposites differing in the cerium/dextran ratio may indicate different intensities of effects on biological objects. It remained to determine the nature of the effect of synthesized nanoparticles on biological objects depending on their physical characteristics and to select the best compounds for further study on animal models.

Several parameters of changes in the physiology and structure of human fibroblasts, the main cells of the skin that provide synthesis of interstitial substance—collagen, which is necessary for wound healing—were taken for such evaluation. Cytotoxicity was determined with a nanocomposite with a maximum concentration of cerium (10^−2^ M). Although all samples showed low toxicity, the best results in terms of safety characteristic were shown by samples with Ce2D and Ce3D ratios, i.e., samples with the minimum, and therefore the most effective, cerium core size. The Ce2D group showed significantly better results both in the parameter of stimulation of fibroblast proliferation in cell culture and in the parameter of their metabolic activity. Thus, the optimal sizes and ratios of cerium core and dextran coating thickness of the synthesized nanocomposite, providing the best conditions for effective cell viability, were determined.

An attempt to obtain a compound that has both a stimulating effect on body cells and antimicrobial activity, which are almost always present in wound contents, may seem like biological nonsense. However, the authors hoped for the antioxidant activity described by some researchers, which is provided by the change in the valence of cerium dioxide depending on the pH of the medium. The use of gas chromatography with spectrometry showed that the best antimicrobial nanocomposites at the time of 24 h of incubation are composites of Ce0.5D and Ce2D; that is, compounds with greater accessibility to microbial bodies and maximally expressed biological activity of nanoceria. Increasing the contamination time to 72 h extinguished this difference between groups and equalized the reported antimicrobial effect. Unfortunately, it was possible to obtain a pronounced but only bacteriostatic effect and not a bactericidal effect. However, it is very likely that with an increase in antimicrobial effects, we would have obtained an increase in toxicity, which would reduce the regenerative functions of the cell culture. At the same time, we have proven the presence of a new type of antimicrobial effect. This is an antimicrobial effect due to redox activity and a change in valence when the pH of the environment in the wound changes (in particular, acidification of the medium in the wound with *E. coli* contamination). This mechanism is fundamentally different from the mechanism of action of antibacterial drugs; therefore, it gives hope that nano-drugs based on cerium dioxide nanoparticles can help in the fight against antibiotic resistance.

## 5. Conclusions

The main conclusions of our work are as follows:

1. The synthesis of sols of nanocomposites—nanocrystalline cerium dioxide less than 5nm in size, coated with polysaccharide dextran, differing in the initial ratio (by mass) of cerium nitrate to dextran, which is reflected in the physicochemical characteristics and final biological properties—was carried out.
2. As the content of dextran in the nanocomposite increases, the particle size of cerium dioxide decreases and the hydrodynamic radius increases.
3. A high level of safety of nanocomposites was proved, while the absence of cytotoxicity (100% cell survival at the maximum tested concentration) was established for Ce2D and Ce3D.
4. Metabolic and proliferative activity of fibroblasts increases as the dextran content increases (and as the size of cerium oxide crystals, the core of nanocomposites, decreases). The lowest level of fibroblast activity was recorded for Ce0.5D and the highest for Ce2D. In all types of nanocomposites, the most effective stimulation of human fibroblasts proliferation was registered at the concentration of 10^−3^ M, with a clear advantage of Ce2D composite, with which the number of fibroblasts was 2.5 times higher than in the control (at 10^−3^ M Ce0.5D—1.4 times, Ce1D—1.5 times, Ce3D—2.0 times relative to the control).
5. The best antimicrobial activity of nanocomposites at a concentration of 10^−2^ M (bacteriostatic action) was found in Ce0.5D and Ce2D, inhibiting the growth and multiplication of *E. coli* after 24 h by an average of 22–27%, and after 48 h, all variants of nanocomposites significantly inhibit the growth and multiplication of *E. coli* by 58–77%, which is most (and equally) expressed in Ce0.5D, Ce1D, and Ce2D, at which *E. coli* multiplication was inhibited equally by an average of three-fold compared to the control groups. After 48 h, all concentrations of Se2D composite showed bacteriostatic activity, inhibiting *E. coli* multiplication by 47–80%, and a dose-dependent effect was determined: the higher the concentration of nano-ceria, the higher the antimicrobial activity.
6. The complex of interdisciplinary studies allowed us to select for further development the Ce2D composite at a concentration of 10^−3^ M, characterized by the balance of simultaneous stimulation of cells with the most predictable dose-dependent effect, including stimulating cell proliferation and metabolism up to 2.5 times and allowing a reduction in the rate of microorganism multiplication by 4 times.

In conclusion, it should be noted that the method of nanocomposite synthesis developed by the authors allows us to talk about the prerequisites for the development of a technique for obtaining for obtaining compounds with predetermined physical, chemical and biological properties. The optimal sizes of the cerium core of the nanocomposite and the thickness of its dextran coating were found. The necessary physical characteristics of the nanocomposite, which provides the best conditions for wound healing biological effects, were determined. The results obtained during the experiment allow us to speak about composites with nanocerium–dextran structure as promising compounds for their usage in medical and veterinary practice for healing and disinfection of skin wounds.

## Figures and Tables

**Table 1:** Study groups for microbiologic examination and sample preparation.

	Groups	Culture Media	Microorganisms	Test Substance	Total Volume
Study groups	Ce0.5De	4.0 mL	0.5 mL of *E. coli* suspension	0.5 mL of Ce0.5D (10^−2^ M) sol	5.0 mL
	Ce1D	4.0 mL	0.5 mL of *E. coli* suspension	0.5 mL of Ce1D (10^−2^ M) sol	5.0 mL
	Ce2D	4.0 mL	0.5 mL of *E. coli* suspension	0.5 mL of Ce2D (10^−2^ M) sol	5.0 mL
	Ce3D	4.0 mL	0.5 mL of *E. coli* suspension	0.5 mL of Ce3D (10^−2^ M) sol	5.0 mL
Control groups	CM + *E. coli*	4.5 mL	0.5 mL of *E. coli* suspension	–	5.0 mL
	CM + *E. coli* + H_2_O	4.0 mL	0.5 mL of *E. coli* suspension	0.5 mL H_2_O	5.0 mL
Comparison groups	CM	5.0 mL	–	–	5.0 mL
	Ceftriaxone	4.0 mL	0.5 mL of *E. coli* suspension	0.5 mL solution	5.0 mL

## Data Availability

The original contributions presented in the study are included in the article, further inquiries can be directed to the corresponding author.
